# Transcriptome of *Aquilaria malaccensis* containing agarwood formed naturally and induced artificially

**DOI:** 10.1186/s13104-021-05532-9

**Published:** 2021-03-25

**Authors:** Farah Adibah Abdul Kadir, Kamalrul Azlan Azizan, Roohaida Othman

**Affiliations:** 1 Department of Biological Sciences and Biotechnology, Faculty of Science and Technology, Universiti Kebangsaan Malaysia, UKM, 43600 Bangi, Selangor Malaysia; 2 Institute of Systems Biology (INBIOSIS), Universiti Kebangsaan Malaysia, UKM, 43600 Bangi, Selangor Malaysia

**Keywords:** *Aquilaria malaccensis*, Agarwood, Transcriptome, Trunk tissues, Natural formation, Artificial induction

## Abstract

**Objectives:**

Agarwood is the aromatic heartwood formed upon wounding of *Aquilaria* trees either naturally formed due to physical wound sustained from natural phenomena followed by microbial infection, or artificially induced using different inoculation methods. Different induction methods produce agarwoods with different aromas which have impacts on their commercial values. In lieu of elucidating the molecular mechanisms of agarwood formation under different treatment conditions, the transcriptome profiles of trunk tissues from healthy *A. malaccensis* tree, and naturally and artificially induced trees were obtained.

**Data description:**

The transcriptome of trunk tissues from healthy *A. malaccensis*, and naturally and artificially induced trees were sequenced using Illumina HiSeq™ 4000 platform which resulted in a total of 38.4 Gb clean reads with Q30 rate of at least 91%. The transcriptome consists of 85,986 unigenes containing 1305 bases on average which were annotated against several databases. From this, 44,654 unigenes were mapped to 290 metabolic pathways in the Kyoto Encyclopedia of Genes and Genomes database. These transcriptome data represent considerable contribution towards *Aquilaria* transcriptome data and enhance current knowledge in comprehending the molecular mechanisms underlying agarwood formation in *Aquilaria* spp.

## Objective

The valuable agarwood is the fragrant resinous heartwood which are important ingredients in fragrances, incense and medicines [1, 2]. Nine *Aquilaria* spp. belonging to the Thymelaeaceae family are agarwood producers with *A. malaccensis* among the primary ones [3, 4]. Agarwood formation is a slow defense response upon wounding of *Aquilaria* trees due to natural phenomena such as gale and insect bites followed by microbial infection [5, 6]. The wood tissues die to avoid damage expansion and form agarwood accompanied by the release of secondary metabolites [7]. The high agarwood demand has caused natural resource depletion of all agarwood-producing *Aquilaria* species and listed them in the Convention on International Trade in Endangered Species of Wild Fauna and Flora Appendix II [8] where stringent jurisdiction controls their trades [9]. *A. malaccensis* is among the eight *Aquilaria* species categorised on the International Union for Conservation of Nature red list as endangered species [10]. Strategies for sufficient supply of agarwood include reforestration and artificial induction method development. Artificial inductions may involve stem wounding alone or coupled with inoculations using microbial cultures and/or chemical stimulants [11]. However, different induction techniques produce agarwoods with different aromas [12, 13] due to different compositions of metabolites released [14–17].

Previous transcriptomic studies have focused on mechanically wounded [18] and chemically induced [19] *A. sinensis*, and *A. malaccensis* senescing calli [20]. Studies on artificially induced *A. malaccensis* had focused on terpene synthase gene expression analyses [21, 22]. There has been no report on transcriptome of *Aquilaria* containing naturally formed agarwood since such trees are rare. To understand the mechanisms of agarwood formation from the different treatments, here we present *A. malaccensis* transcriptomes from healthy, and naturally formed and artificially induced trees. The annotated transcriptomes presented here provide a valuable resource for researchers interested in agarwood formation in agarwood-producing species.

## Data description

Identification of *A. malaccensis* Lam. [23] was performed by A. Damanhuri (Curator for Universiti Kebangsaan Malaysia Herbarium; UKMB) for voucher specimen labelled as M. H. Azhari 1. Trunk samples from uninjured trees were used as control, whilst agarwood-containing trunk samples were obtained from naturally and artificially induced trees. Naturally formed agarwood was found in broken tree trunks resulting from natural phenomena combined with microbial infection. Artificially induced agarwood was formed 5 years after nail wounding combined with honey-containing inoculum injection.

### RNA extraction, library construction and sequencing

Total RNA extracted following the Trizol protocol [24] was evaluated for quantity and quality using NanoDrop spectrophotometer (Thermo Fisher Scientific Inc., USA) and Agilent 2100 Bioanalyzer (Agilent Technologies, USA). Total RNAs having RNA integrity number (RIN) values of at least 8.0 were used for construction three cDNA libraries using commercial service provided by Macrogen, Inc. (Seoul, Republic of Korea) on Illumina HiSeq 4000 platform (San Diego, USA) (Datasets 1–3). A schematic overview of this study is shown in Data file 1.

### Transcriptome assembly

Raw reads (271,072,542) obtained through Illumina HiSeq sequencing were filtered to remove reads with low quality (Data file 5). Adapter sequences in the raw reads were eliminated using Cutadapt software (version 2.3–0) and raw reads quality was examined using FastQC version 0.11.2 [25]. High quality reads (Data file 5) were acquired by removing adapters and other undesirable sequences using Trimmomatic [26]. Trinity version 2.1.1 [27] was utilised for the assembly of the reads de novo [28] while the reads were clustered into non-redundant unigenes set using TIGR Gene Indices clustering tools version 2.1 [29]. Transcript abundance was calculated by mapping the trimmed reads on to the assembled transcripts and measured with RNA-Seq by employing Expectation Maximization (RSEM) version 1.2.196. From the output, transcripts with the values of Fragments Per Kilobase of exon Per Million fragments mapped (FPKM) less than one were filtered from the original transcripts file to produce the final unigenes consisting of 85,986 contigs (Data file 5). The contig lengths of *A. malaccensis* ranged from 201 to 25,238 bp with 1305 bp average length. BLAST searches were conducted for gene functional annotations against public databases such as Swiss-Prot, GO (Gene Ontology) and KEGG (Kyoto Encyclopedia of Genes and Genomics) (Data files 2, 3 and 6). The annotated unigenes were mapped to KEGG database to hit a total of 290 metabolic pathways (Data file 4).

### Quality, completeness and depth of the *A. malaccensis* transcriptome

The *A. malaccensis* transcriptome was appraised using BUSCO (Benchmarking Universal Single-Copy Orthologs) to determine the completeness of the unigene assembly [30]. Embryophyta and eudicotyledon gene sets were used which have 1375 and 212 near-universal single-copy orthologs, respectively (Data file 7). Comparison of the statistics for *A. malaccensis* transcriptome and the previously published *A. sinensis* transcriptomes [18, 19] showed 238 and fivefold increases of the former over the 454-based [18] and Ilumina-based *A. sinensis* transcriptomes [19], respectively (Data file 8). The *A. malaccensis* high quality reads also have the highest average transcript lengths of 1305 bp (see Table 1).

**Table 1:** Overview of data files/data sets

Label	Name of data file/data set	File types (file extension)	Data repository and identifier (DOI or accession number)
Data set 1	RNA-Seq of healthy trunk tissue of *Aquilaria malaccensis*	fastq file (.fastq)	NCBI sequence read archive (https://identifiers.org/insdc.sra:SRR8863603) [31]
Data set 2	RNA-Seq of naturally induced agarwood from trunk tissue of *Aquilaria malaccensis*	fastq file (.fastq)	NCBI sequence read archive (https://identifiers.org/insdc.sra:SRR8863602) [32]
Data set 3	RNA-Seq of synthetically induced agarwood from trunk tissue of *Aquilaria malaccensis*	fastq file (.fastq)	NCBI sequence read archive (https://identifiers.org/insdc.sra:SRR8863604) [33]
Data set 4	TSA: *Aquilaria malaccensis*, transcriptome shotgun assembly	Entry note	NCBI Sequence Read Archive https://identifiers.org/ncbi/insdc:GHJS00000000.1 [34]
Data file 1	Schematic overview of the study	Image file (.tiff)	Figshare (https://doi.org/10.6084/m9.figshare.13417400.v2) [35]
Data file 2	Comparison of functional annotations for *Aquilaria malaccensis* unigenes against different databases	Image file (.tiff)	Figshare (https://doi.org/10.6084/m9.figshare.13417400.v2) [35]
Data file 3	Functional classifications of *Aquilaria malaccensis* unigenes	Image file (.tiff)	Figshare (https://doi.org/10.6084/m9.figshare.13417400.v2) [35]
Data file 4	Most enriched KEGG pathways for *A. malaccensis* differentially expressed genes	Document file (.docx)	Figshare (https://doi.org/10.6084/m9.figshare.13417400.v2) [35]
Data file 5	Summary statistics for transcriptome assembly and annotations sequencing results for *Aquilaria malaccensis*	Document file (.docx)	Figshare (https://doi.org/10.6084/m9.figshare.13417400.v2) [35]
Data file 6	Summary of gene functional annotations for *Aquilaria malaccensis* unigenes	Document file (.docx)	Figshare (https://doi.org/10.6084/m9.figshare.13417400.v2) [35]
Data file 7	BUSCO analysis of assembly completeness	Document file (.docx)	Figshare (https://doi.org/10.6084/m9.figshare.13417400.v2) [35]
Data file 8	Comparison of transcriptome assembly and annotation statistics for *Aquilaria* spp.	Document file (.docx)	Figshare (https://doi.org/10.6084/m9.figshare.13417400.v2) [35]

## Limitations

All the samples used were of different ages. The uninjured healthy tree was much younger than the trees containing agarwood. The trees containing naturally formed agarwood were much older since the process of agarwood formation took much longer than 5 years which was the time period for the formation of agarwood in the artificially induced trees.

## Data Availability

The raw fastq files were deposited in the National Center for Biotechnology Information and are available with accession numbers (SRR8863602–SRR8863604) under Bioproject PRJNA497968 (Datasets 1–3); SRR863602, SRR8863603, SRR8863604) [31–33]. Assembly of non-redundant ORF unigene sequences are available from NCBI transcriptome shotgun assembly (TSA) database (https://identifiers.org/ncbi/insdc:GHJS00000000.1) [34]. The supplementary materials (Figures S1–S3, Tables S1–S5) can be accessed on Figshare (https://doi.org/10.6084/m9.figshare.13417400.v2) [35]. Please see Table 1 and reference list for details and links to the data.

## Abbreviations

IUCN: International Union for Conservation of Nature
CITES: Convention on International Trade in Endangered Species of Wild Fauna and Flora
FPKM: Fragments Per Kilobase of exon Per Million fragments mapped
GO: Gene ontology
KEGG: Kyoto Encyclopedia of Genes and Genomics
BUSCO: Bench-marking universal single-copy ortholog
SRA: Short read archive
TSA: Transcriptome shotgun assembly
